# Ganoderic Acid A Attenuates LPS-Induced Neuroinflammation in BV2 Microglia by Activating Farnesoid X Receptor

**DOI:** 10.1007/s11064-021-03303-3

**Published:** 2021-04-05

**Authors:** Yue Jia, Dandan Zhang, Hua Yin, Haoran Li, Jing Du, Hongkun Bao

**Affiliations:** 1 School of Medicine, Yunnan University, 2 Cuihu North Road, Kunming, 650091 Yunnan People’s Republic of China; 2 The National Clinical Research Center for Mental Disorders and Beijing Key Laboratory of Mental Disorders, Beijing Anding Hospital, Capital Medical University, Beijing, 100088 People’s Republic of China; 3 Advanced Innovation Center for Human Brain Protection, Capital Medical University, Beijing, 100088 People’s Republic of China; 4 Yunnan Key Laboratory of Molecular Biology of Chinese Medicine, Yunnan University of Traditional Chinese Medicine, Kunming, 650500 Yunnan People’s Republic of China

**Keywords:** Neuroinflammation, Ganoderic Acid A, BV2 Microglia, FXR

## Abstract

Neuroinflammation plays an important role in the onset and progression of neurodegenerative diseases. Microglia-mediated neuroinflammation have been proved to be the main reason for causing the neurodegenerative diseases. Ganoderic acid A (GAA), isolated from *Ganoderma lucidum*, showed anti-inflammatory effect in metabolism diseases. However, little research has been focused on the effect of GAA in neuroinflammation and the related mechanism. In the present study, lipopolysaccharide(LPS)-stimulated BV2 microglial cells were used to evaluate the anti-inflammatory capacity of GAA. Our data showed that GAA significantly suppressed LPS-induced BV2 microglial cells proliferation and activation in vitro. More strikingly, GAA promoted the conversion of BV2 microglial cells from M1 status induced by LPS to M2 status. Furthermore, GAA inhibited the pro-inflammatory cytokines release and promoted neurotrophic factor BDNF expression in LPS-induced BV2 microglial cells. Finally, we found that the expression of farnesoid-X-receptor (FXR) was prominently downregulated in LPS-stimulated BV2 microglial cells, antagonism of FXR with z-gugglesterone and FXR siRNA can reverse the effect of GAA in LPS-induced BV2 microglial cells. Taking together, our findings demonstrate that GAA can significantly inhibit LPS-induced neuroinflammation in BV2 microglial cells via activating FXR receptor.

## Introduction

Neuroinflammation, inflammation of the central nervous system (CNS), is an immune response often initiated against a variety of harmful stimuli, including pathogens, trauma and neural damage, etc. Accumulative evidence strongly suggested that neuroinflammation is a common feature of neurodegenerative diseases, such as Parkinson’s diseases (PD), multiple sclerosis (MS) and Alzheimer’s diseases (AD), and is associated with the progressive loss of neuronal structure and function [1–3]. The inflammation reaction is an automatic defense response of the body to external stimuli. In some cases, it is usually beneficial because it can promote the clearance of pathogenic factors and the healing of damaged tissue; but in other cases, it is detrimental because it can aggravate the damage of injured tissue or cells and worsen the condition [4]. The strategies to modulate the inflammatory processes are increasingly considered as the candidate options to therapy inflammation related disease.

Microglia are the resident macrophages of the CNS and plays an important role in immune surveillance, homeostasis and neuroinflammation [5]. Under normal conditions, microglia not only provide immune surveillance but also respond to harmful stimuli; under pathologic conditions, microglia can be activated in order to respond to the detrimental signals. Similar to macrophages, microglia was heterogeneous [6]. Generally speaking, activated microglia can be categorized as the classic pro-inflammatory M1 type or the anti-inflammatory M2 type. M1 type microglia were characterized by an overproduction of inflammatory cytokines and inflammatory mediators, including tumor necrosis factor(TNF)-α, interleukin(IL)-6, IL-1β, inducible nitric oxide synthase(iNOS) and prostaglandin G2(PG2), etc., [7]. On the contrary, M2 type microglia were characterized by the secretion of anti-inflammatory cytokines including IL-4, IL-10 and transforming growth factor(TGF)-β [8]. M1 type microglia play a detrimental effect while M2 type microglia exert a neuroprotective and regenerative effect. Therefore, it is of great importance to regulate the differentiation of microglia and reduce the inflammatory damage.

Taking these factors into consideration, researchers focus their interest on natural products with potential anti-inflammatory and neuroprotective effects. Previous studies have discovered many natural products, which can converse the polarization of microglia from M1 to M2 in vitro and in vivo [9–11]. Ganoderic acid A (GAA), isolated from Ganoderma lucidum, is proved to exert anti-tumor, anti-oxidant, anti-inflammatory and hepatoprotective effects [12–14]. The protective role of Ganoderma lucidum extracts on neurons has also been well studied [15]. However, the specific effect of GAA on neuroinflammation remains unknown, even though GAA is a major pharmaceutically active compound of Ganoderma lucidum. Based on these findings, we hypothesize that GAA has an inhibitory effect on neuroinflammation and can interfere with microglial polarization.

The farnesoid-X-receptor (FXR, NR1H4), also known as a bile acid receptor, was a ligand-activated transcriptional factor and belongs to the nuclear hormone receptor superfamily. FXR has been extensively studied in human metabolic disease [16, 17]. Recently, the researcher found that FXR plays a neuroprotective role in multiple sclerosis [18]. Growing evidence indicated that GAA can activate FXR [19]. However, whether GAA can inhibit inflammation via activation FXR, it remains unclear. Therefore, this study aimed to investigate the effects of GAA on LPS-induced inflammation of microglial cells and to explore the involved mechanisms.

## Materials and Methods

### Materials

**Fig. 1:**
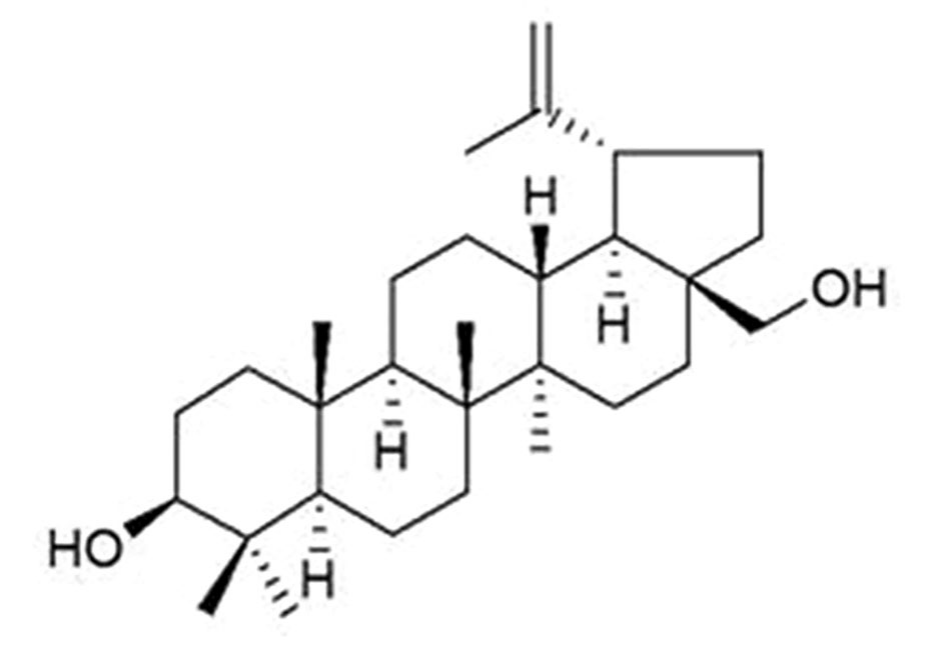
Chemical structure of Ganoderic acid A

GAA (Cat: B20742) was purchased from Shanghai Yuanye Biology Co. (Shanghai, China), the molecular structure of GAA was shown as Fig. 1. LPS (Cat: L2630) and Z-gugglesterone (GS) (Cat: 370690) were purchased from sigma. CCK-8 Kit (Cat:BS350B) was purchased from biosharp Life Sciences. Mouse IL-1β (Cat: ab197742) and TNF-α (Cat: ab208348) ELISA kits were purchased from Abcam. Mouse IL-6 (Cat: VAL604) and BDNF (Cat: VAL136) ELISA kits were purchased from NOVUS. The primary antibodies, including anti-ionized calcium-binding adapter molecule 1(Iba1) (Cat: ab5076), anti-iNOS (Cat: ab15323), anti-arginase(Arg)-1 (Cat: ab91279), anti-FXR (Cat: ab85606), anti-IL-6 (Cat: ab208113), anti-brain derived neurotrophic factor(BDNF) (Cat: ab108319) and anti-GAPDH (Cat: ab8245) were purchased from Abcam, anti-IL-1β (Cat: AF-401-NA) and anti-TNF-α (Cat: AF-410-NA) were purchased from R&D, anti-beta Tubulin (Cat:MA5-11732) was purchased from Thermo Scientific. The second antibodies, including Alexa Fluor488 labeled Bovine Anti-Goat (Cat: 805–545-180) and Alexa Fluor 594 (Cat: 711-585-152) labeled Donkey anti-Rabbit were purchased from Jackson Labs. Donkey anti-Goat IgG (H + L) HRP (Cat: A15999) was purchased from Invitrogen. Goat anti-Rabbit IgG (H + L) HRP (Cat: S0001) and Goat anti-Mouse IgG (H + L) HRP (Cat: S0002) were purchased from Affinity. DAPI (Cat: 36308ES11) was purchased from Yeasen Biotech Co., Ltd.

### Cell Culture

Murine BV2 microglial cell line was provided by Dr. Qi Yan, Yunnan University of Traditional Chinese Medicine. The cells were cultured in DMEM high glucose complete medium (Cat: 10-013-CVRC), supplemented with 10% fetal bovine serum (FBS) (Cat: 04-0011-1ACS) and 1% penicillin streptomycin solution at 37 °C in a humidified incubator under 5% CO_2_ in T25 flasks. When reached over 80% confluence, cells were seeded onto 96-well, 24-well or 6-well plate for further experiments.

### Cell Counting Kit-8 Assay

BV2 microglial cells were plated in 96-well plates at a density of 5 × 10^3^ cells per well, all of the study was conducted 24 h after the cells were seeded. Cells were then treated with LPS (0.1, 0.25, 0.5, 0.75, 1 and 2 μg/ml), GAA (1, 25, 50, 75, 100 and 200 μg/ml) and GS (10, 20, 40, 60, 80 and 100 μM) for 24 h. After treatment, the cells were rinsed with PBS for twice and the medium was changed to 100 µl DMEM high glucose medium and 10 μl of CCK-8 was added into the culture plate. Followed by incubation at 37 °C for 2 h, the optical density value at the wavelength of 450 nm was detected by using a microplate reader (Epoch, BioTek Instruments, Winooski, USA). After correction by subtracting the optical density value of wells that did not contain cells, experimental data were shown as relative cell viability normalized to the control group [13].

### Drug Treatments

BV2 microglial cells were stimulated with 0.5 μg/ml LPS as an inflammation state in vitro. For the study of GAA on LPS induced neuroinflammation, GAA was administrated in simultaneously with LPS to the BV2 cells. After 24 h treatment, cytokines, FXR and microglial biomarker were detected by western blot and immunofluorescence methods.

For the study of GS (a selective FXR receptor antagonist) on GAA and LPS co-treated BV2 microglial cells, GS was administrated to BV2 microglial cells 2 h before LPS and GAA treatment. After 24 h treatment, cytokines were detected by western blot method.

### Transient Transfection with siRNA

When BV2 microglial cells were confluent to 60–70%, they were transfected with FXR siRNA (1.5 μg) or negative control siRNA (1.5 μg) using the DNAfectin™ Plus Transfection Reagent (Cat: G2500, Applied Biological Materials Inc). The siRNA sequence targeting FXR 5′-GGCGUAGCAUUACCAAGAATT-3′ was designed and supplied by GenePharma. After 36 h, the DNAfectin™ Plus Transfection Reagent were removed and the cells were treated with GAA and LPS. 24 h later, the inhibition of siRNA on FXR expression and the expression of TNF-α and BDNF in BV2 microglial cells were detected by western blot.

### ELISA for IL-1β, IL-6, TNF-α and BDNF

After 24 h treatment, the levels of IL-1β, IL-6, TNF-α and BDNF in cell culture supernatant were measured according to manufacturer's instructions using ELISA kits. Results were expressed as pg/ml of supernatant.

### Western Blot

After 24 h treatment, the cell culture medium was discarded and the cells were washed three times with ice-cold PBS. 200 μl of RIPA cell lysis buffer mixed with protease and phosphatase inhibitors were added to each well, then cells were incubated on ice for 30 min, and the lysate was collected by spinning at the speed of 12,000 rpm for 10 min at 4 °C, the supernatants were used for following study. Protein concentrations were determined using a BCA protein assay kit (Pierce Biotechnology, Rockford, USA). Equal amounts of proteins were subjected to 10–12.5% SDS-PAGE gel electrophoresis and transferred to 0.22 µm polyvinylidene difluoride (PVDF) membranes (Cat: ISEQ00010, Merck Millipore Ltd). Antibodies against Iba1, iNOS, Arg-1, IL-1β, IL-6, TNF-α, BDNF and FXR were used as primary antibodies. Secondary antibodies, including Donkey anti-Goat IgG (H + L) HRP, Goat anti-Rabbit IgG (H + L) HRP and Goat anti-Mouse IgG (H + L) HRP. The anti-GAPDH and anti-Tubulin antibodies were applied for loading calibration. Immunoreactive bands were visualized using the ECL detection system (Millipore, Billerica, USA). The images were acquired by the chemiluminescent imaging system (Amersham Imager 600, GE) and quantified using Image Pro Plus version 6.0 software (Media Cybernetics, Rockville, USA).

### Immunofluorescent Staining

After LPS and GAA treatments, cultured BV2 microglial cells were washed thrice with cold 1 × PBS and fixed in 4% paraformaldehyde in PBS for 20 min at room temperature. The cells were then incubated with blocking buffer (1% BSA and 0.2% Triton X-100 in PBS) for 1 h at room temperature. Next, cells were incubated with primary anti-Iba1, anti-iNOS, anti-Arg1 and anti-FXR antibodies at 4 °C overnight. Cells were then washed with PBST for three times, appropriate secondary antibodies labeled with Alexa Fluor 488 or Alexa Fluor 594 was prepared in PBST containing 5% BSA. After washing, cells were incubated with second antibody solution for 1 h at room temperature and rinsed with PBST thrice. After washing, the cells were mounted onto slides with anti-fade mounting media containing DAPI solution.

### Image Analysis

All slides were photographed and digitized using a video camera mounted on a Leica microscope (Leica DM2500, Germany). All images were taken under exactly the same conditions, including laser output strength, exposure time, gain, offset, etc. BV2 microglial cells were randomly photographed, with 5 or more images obtained for each coverslip to ensure that conditions for each coverslip in each treatment group were the same. Pictures were further processed using Adobe Photoshop CS5 (Adobe Systems Software, Ireland).

### Statistical Analysis

All data were analyzed with one-way ANOVA followed by Turkey post hoc test. All data were analyzed using Graph Pad Prism Ver. 5.0 (Graph Pad Software, Inc., San Diego, CA) and expressed as the mean ± SEM. *P* values less than 0.05 were considered statistically significant. Figures were generated by GraphPad Prism version 5 software.

## Results

### GAA Inhibits LPS-Induced BV2 Microglial Cells Proliferation and Activation In Vitro

**Fig. 2:**
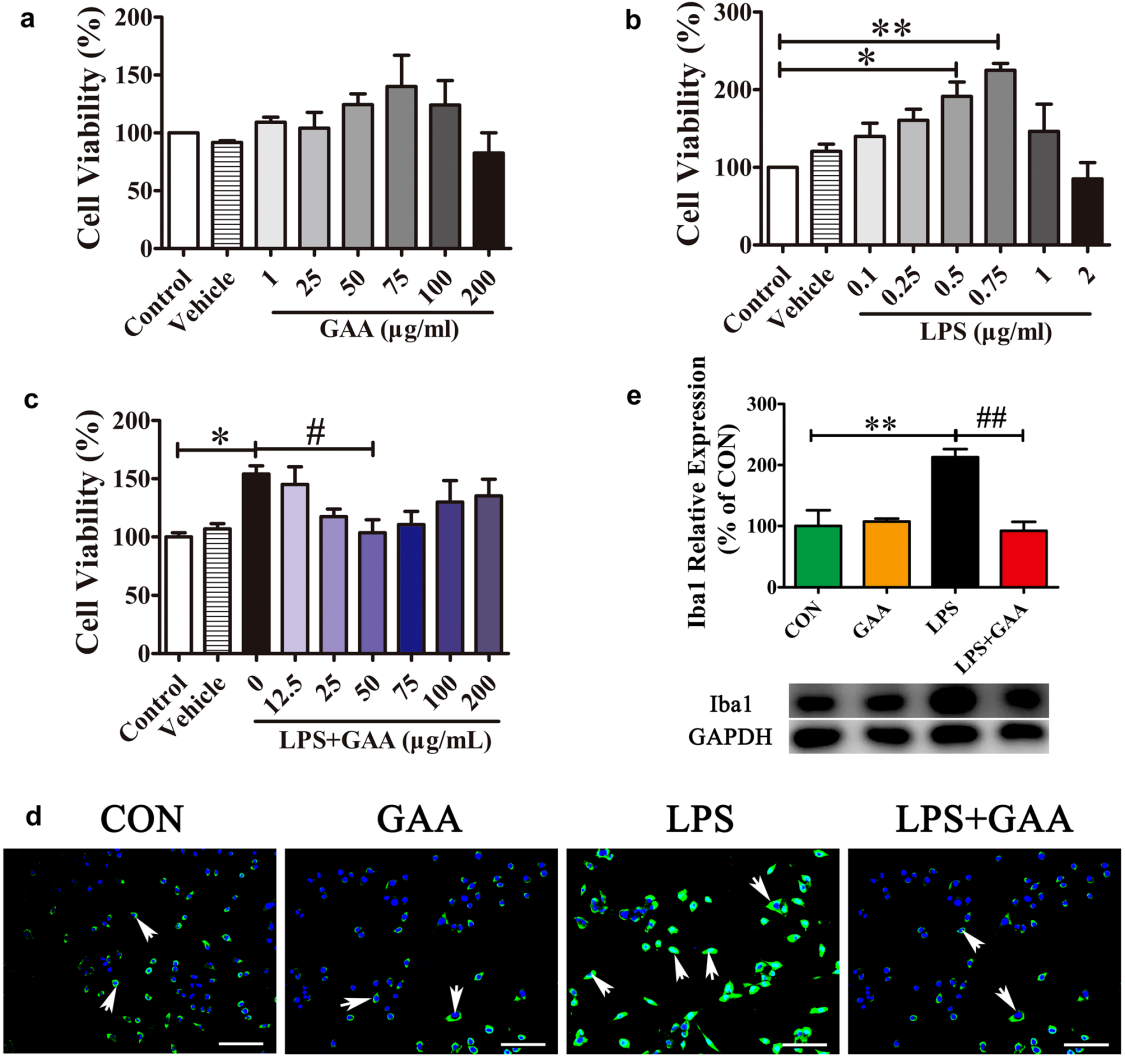
GAA suppressed the LPS-induced BV2 microglial cells proliferation and activation in vitro. **a** BV2 cells were cultured with different concentration of GAA for 24 h. **b** BV2 cells were stimulated with different concentration of LPS for 24 h. **c** BV2 cells were cultured with different concentration of GAA in the presence of 0.5 μg/ml LPS for 24 h. Cell proliferation was detected by CCK-8 assay. **d** Immunofluorescence images showing the BV2 microglial cells after LPS stimulation which was labeled with anti-Iba1 antibody, With GAA, the expression of Iba1 is decreased. Scale bar equals to 100 μm. **e** The protein levels of Iba1 were detected by Western blot. After normalization to the control, data were analyzed using one-way ANOVA followed by post hoc Turkey tests and were presented as Mean ± SEM for three independent experiments. (**a**–**c** *P < 0.05 LPS 0.5 µg/ml vs. CON, ^**^P < 0.01 LPS 0.75 µg/ml vs. CON, ^#^*P* < 0.05 LPS + GAA 50 µg/ml vs. LPS; Fig. 1e, **P < 0.01 LPS vs. CON; ^##^*P* < 0.01 LPS + GAA vs. LPS)

To evaluate the potential cytotoxicity of GAA in BV2 microglial cells, the BV2 microglial cells were treated with different dosages of GAA (1, 25, 50, 75, 100, 200 μg/ml) for 24 h. Cell viability was measured using the CCK-8 kit. The results showed that GAA treatment had no cytotoxicity to BV2 microglial cells at the dosage from 1 to 100 μg/ml, it began to exert cytotoxic effect at the dosage of 200 μg/ml (Fig. 2a). Furthermore, as we used LPS to induce microglia inflammation, it was necessary to ascertain the dosage of LPS that had no cytotoxicity. We found that low dose of LPS had no cytotoxic effect to BV2 cells, but high dosage of LPS (2 μg/ml) began to exert cytotoxic effect. What is more important, we found that LPS can promote BV2 cell proliferation in vitro at concentration of 0.5 μg/ml and 0.75 μg/ml (Fig. 2b). Following, we evaluated whether GAA can inhibit LPS-induced microglial proliferation, BV2 microglial cells were cultured with different concentrations of GAA and with LPS (0.5 μg/ml) for 24 h. The results showed that GAA (50 μg/ml) inhibited LPS-induced BV2 microglial proliferation (Fig. 2c). Thus, we selected the effective dose of 50 μg/ml GAA and 0.5 μg/ml LPS for further study. Cellular immunofluorescence showed that the BV2 microglial cells were activated in response to LPS stimulation, the cell bodies became larger and rounder. Iba1 is a microglia/macrophage-specific calcium-binding protein and is often used to evaluating microglia activation [20]. As compared to the control group, the expression of Iba1 was upregulated obviously. As compared to LPS group, GAA treatment can significantly suppress the expression of Iba1 (Fig. 2d). The results were also confirmed by western blotting method. GAA can significantly decrease the expression of Iba1 after LPS stimulation (Fig. 2e).

### GAA Promoted the Conversion of LPS-Induced Microglial Cells from M1 Status to M2 Status

**Fig. 3:**
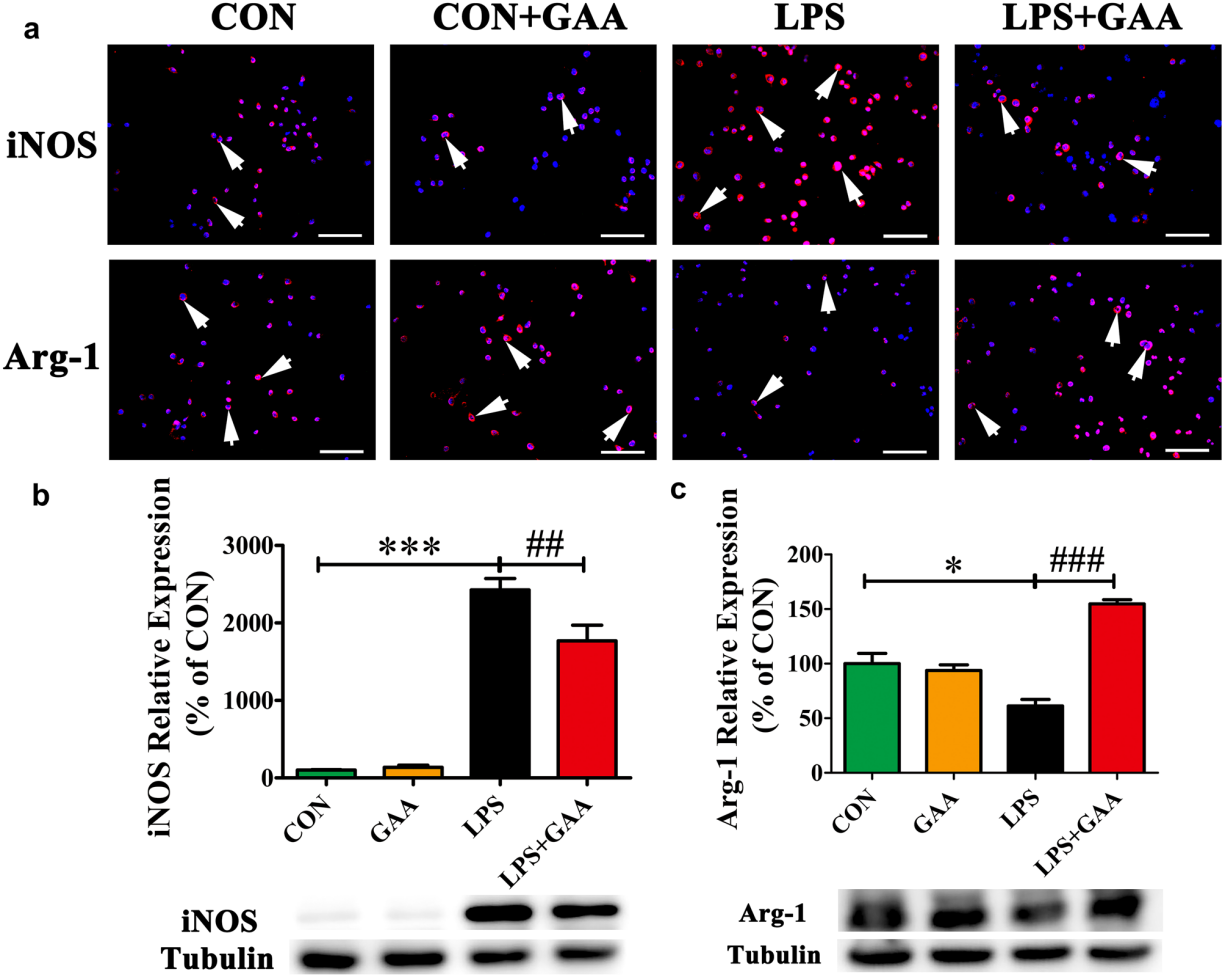
GAA suppressed the up-regulation of iNOS and the down-regulation of Arg-1 in LPS-stimulated BV2 microglial cells. **a** Immunofluorescence images showing the BV2 microglial cells after LPS stimulation which were labeled with anti-iNOS or anti-Arg-1 antibody. With GAA, the expression of iNOS was decreased and the expression of Arg-1 was increased. Scale bar equals to 100 μm. **b** The protein levels of iNOS were detected by Western blot. **c** The protein levels of Arg-1 were detected by Western blot. After normalization to the control, data from three independent experiments was analyzed using one-way ANOVA followed by post hoc Turkey tests and were presented as Mean ± SEM. (*P < 0.05, ***P < 0.001 LPS vs. CON; ^##^*P* < 0.01, ^###^*P* < 0.001 LPS + GAA vs. LPS)

Reactive polarized microglia were characterized by differential expression of cell surface markers. To evaluate M1/M2 polarization, we analyzed the expression of M1 and M2 cell surface markers (iNOS and Arg-1 respectively) in LPS-induced BV2 microglial cells after GAA treatment for 24 h [21]. The results showed that iNOS was significantly increased after LPS stimulation, GAA treatment could inhibit the up-regulation of iNOS in LPS-induced BV2 microglial cells. On the contrary, the expression of Arg-1 was significantly decreased after LPS stimulation, GAA treatment significantly reversed the down-regulation of Arg-1 in LPS-induced BV2 microglial cells (Fig. 3a). In order to further confirm this, we measured the expression iNOS and Arg-1 using western blot. The results showed that GAA reversed the up-regulation of iNOS and the down-regulation of Arg-1 in LPS-induced BV2 microglial cells (Fig. 3b, c). In other words, GAA promoted the shift of M1 status to M2 status in LPS-induced BV2 microglial cells.

### GAA Attenuated Pro-Inflammatory Cytokines IL-1β, IL-6 and TNF-α and Enhanced Neurotrophic Factor BDNF Expression in LPS-Induced BV2 Microglial Cells

**Fig. 4:**
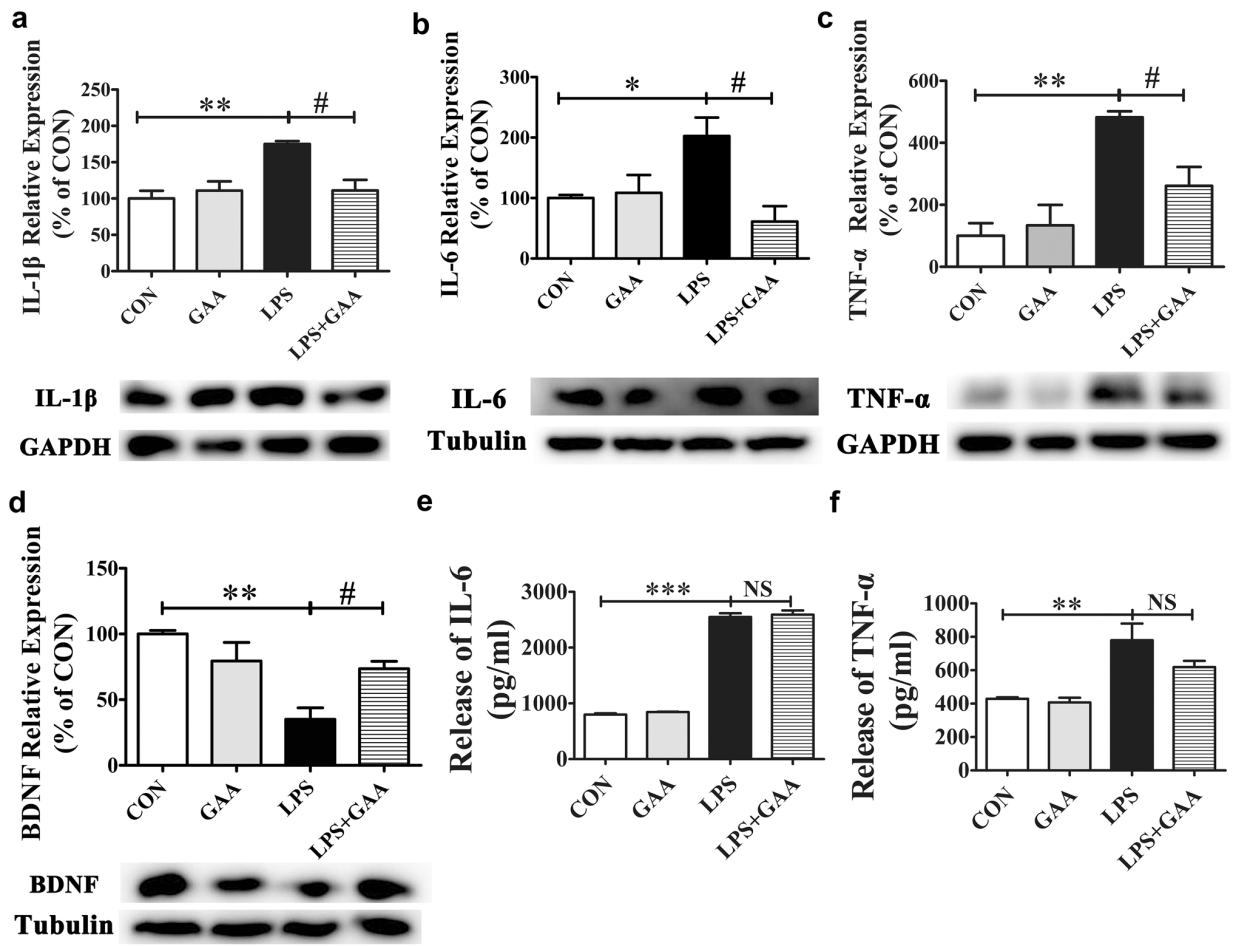
The effects of GAA on IL-1β, IL-6, TNF-α and BDNF expression levels in LPS-stimulated BV2 microglial cells. The protein levels of IL-1β (**a**), IL-6 (**b**), TNF-α (**c**) and BDNF (**d**) were detected by Western blot. After normalization to the control, data from three independent experiments were analyzed using one-way ANOVA followed by post hoc Turkey tests and were presented as Mean ± SEM. The protein levels of IL-6 (**e**) and TNF-α (**f**) were detected by ELISA assay. Data were analyzed using one-way ANOVA followed by post hoc Turkey tests and were presented as Mean ± SEM. N = 5–6 each group. (*P < 0.05, **P < 0.01, ***P < 0.01 LPS vs. CON; ^#^*P* < 0.05 LPS + GAA vs. LPS)

It has been reported that LPS stimulation induced inflammatory response in microglial cells, resulting in the release of pro-inflammatory cytokines. In order to evaluate the effect of GAA on the production of LPS-induced pro-inflammatory cytokines, we detected the levels of TNF-α, IL-1β and IL-6 in cell lysates using the western blot method. The results showed that compared with the control group, the expression of TNF-α, IL-1β and IL-6 were significantly increased after LPS stimulation. However, GAA treatment significantly inhibited the LPS-induced TNF-α, IL-1β and IL-6 secretion in BV2 cells (Fig. 4a–c). In addition, BDNF plays an important role in anti-inflammatory effects, we measured the expression of BDNF after LPS stimulation and found that the expression of BDNF was also significantly decreased by 64.9% (*P* < 0.01) after LPS stimulation. However, GAA treatment significantly reversed LPS-induced BDNF down-regulation by 38.5% (*P* < 0.05) (Fig. 4d). Furthermore, we detected the levels of TNF-α, IL-1β, IL-6 and BDNF in cell culture supernatant using ELISA method. The results showed that the expression of TNF-α and IL-6 were significantly increased in the cell culture supernatants of LPS-stimulated BV2 microglial cells compared with control group, but GAA treatment could not significantly decrease the expression of TNF-α and IL-6 in the cell culture supernatants of LPS-stimulated BV2 microglial cells (Fig. 4e, f). Unfortunately, we did not detect the expression of IL-1β and BDNF in the supernatants of LPS-stimulated BV2 microglial cells according to the manufacturer’s instructions. These results further confirmed that GAA can promote the conversion of BV2 microglial cells from M1 to M2 after LPS stimulation.

### GAA Reversed LPS-Induced FXR Downregulation in BV2 Cells

**Fig. 5:**
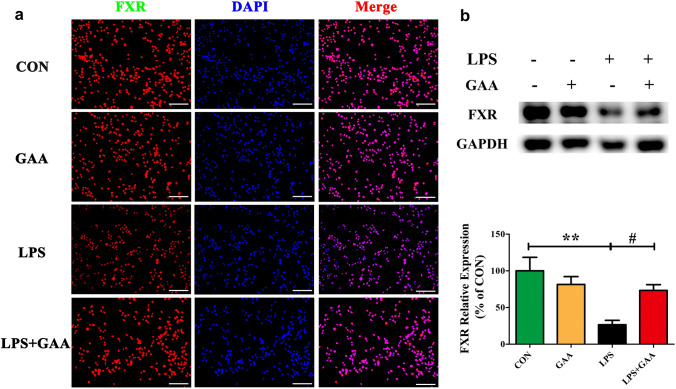
GAA reversed the down-regulation of FXR in LPS-stimulated BV2 microglial cells. **a** Immunofluorescence images showing the BV2 microglial cells after LPS stimulation which were labeled with anti-FXR antibody. With GAA, the expression of FXR is significantly up-regulated. Scale bar equals to 100 μm. **b** The protein levels of FXR were detected by Western blot. After normalization to the control, data from three independent experiments was analyzed using one-way ANOVA followed by post hoc Turkey tests and were presented as Mean ± SEM. (**P < 0.05 LPS vs. CON; ^#^*P* < 0.05 LPS vs. LPS + GAA)

To further ascertain the mechanism of the GAA effects in BV2 microglial cells after LPS stimulation, the effects of GAA on FXR expression were detected. Cellular immunofluorescence staining showed that FXR was significantly decreased after LPS stimulation, GAA treatment reversed the FXR down-regulation after LPS stimulation (Fig. 5a). In order to further confirm the data, we measured the expression of FXR by western blot. The results showed that GAA treatment can increase the expression of FXR by 46.7% (*P* < 0.05) after LPS stimulation (Fig. 5b), which was consistent with the immunostaining results.

### GS, A Selective FXR Antagonist, Blocked the Effects of GAA in LPS-Stimulated BV2 Microglial Cells

**Fig. 6:**
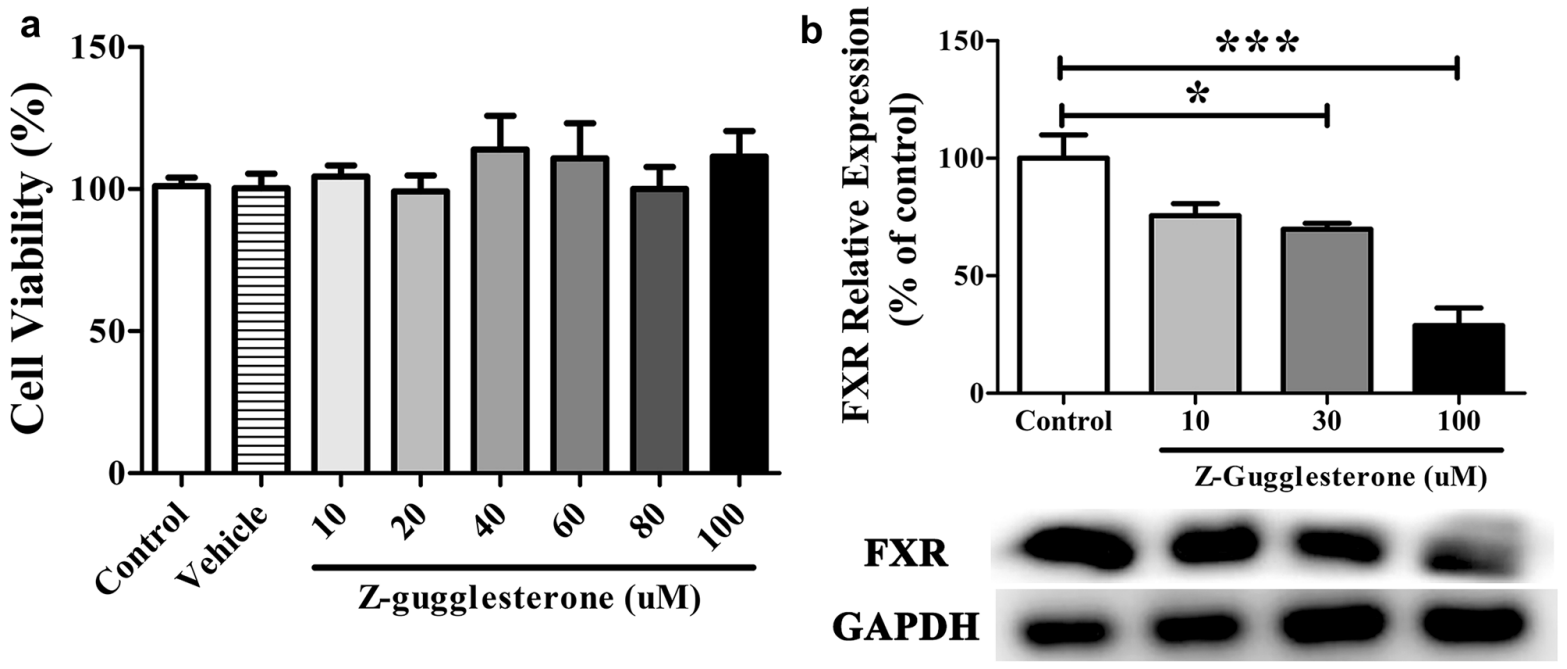
GS dose-dependently blocked the expression of FXR in LPS-stimulated BV2 cells. **a** BV2 cells were stimulated with different concentration of GS for 24 h. **b** The protein levels of FXR were detected by Western blot. After normalization to the control, data from three independent experiments was analyzed using one-way ANOVA followed by post hoc Turkey tests and were presented as Mean ± SEM. (*P < 0.05 GS 30 μM vs. control; ***P < 0.001 GS 100 μM vs. control)

**Fig. 7:**
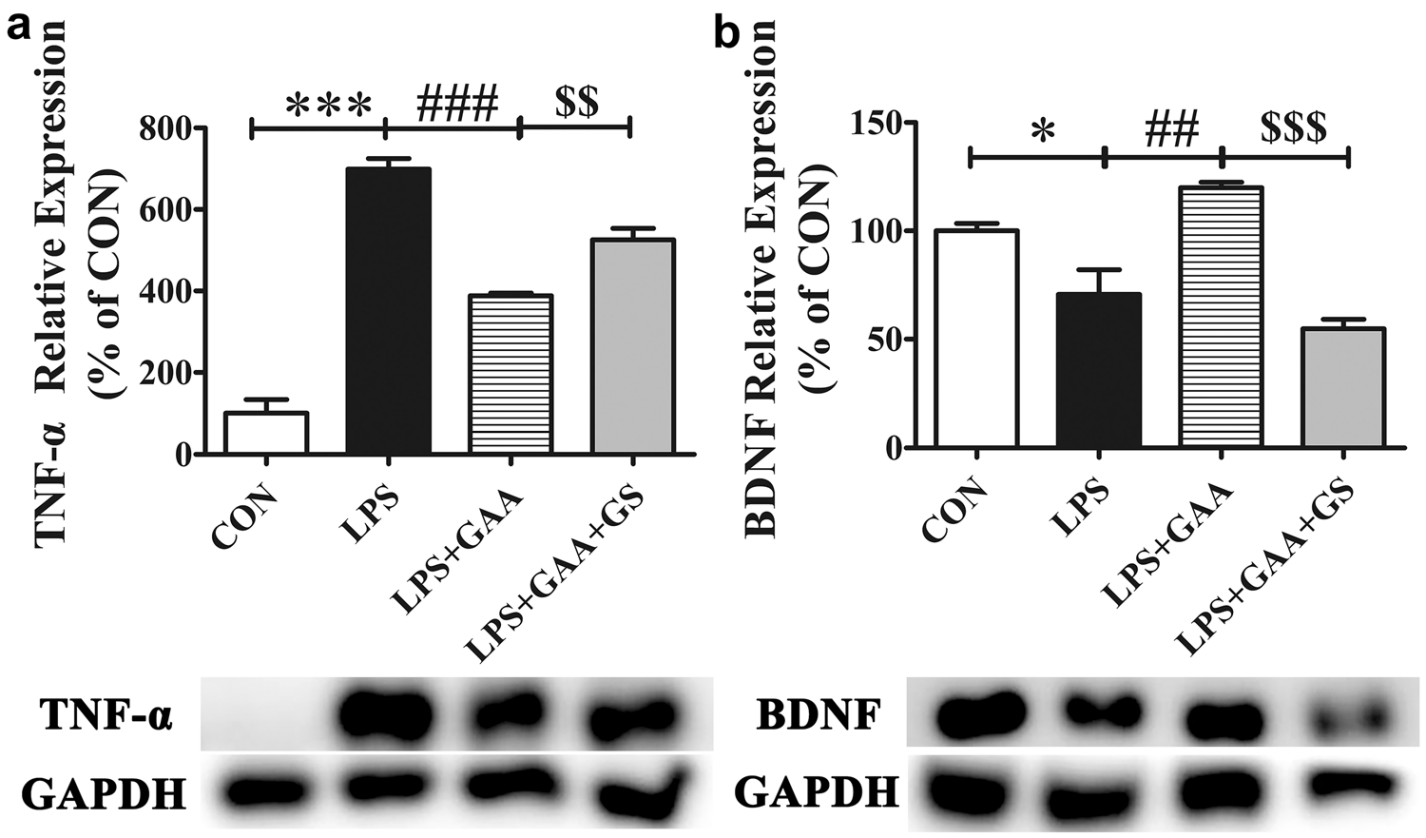
GS inhibited the anti-inflammatory effects of GAA in LPS-stimulated BV2 microglial cells. The protein levels of TNF-α (**a**) and BDNF (**b**) were detected by Western blot. GS was administrated to BV2 microglial cells for 2 h before LPS and GAA treatment for 24 h. After normalization to the CON, data from three independent experiments was analyzed using one-way ANOVA followed by post hoc Turkey tests and were presented as Mean ± SEM. (*P < 0.05*,* ***P < 0.001 LPS vs. CON; ^##^*P* < 0.01*,* ^###^*P* < 0.001 LPS + GAA vs. LPS; ^*$$*^*P* < 0.01, ^*$$$*^*P* < 0.001 LPS + GAA + GS vs. LPS + GAA)

To further evaluate the modulation mechanism of GAA in LPS-induced BV2 microglial cells, GS, a selective FXR antagonist was used to block FXR. First, we evaluated the cytotoxic effect of GS to BV2 microglial cells. The results showed that GS had no cytotoxicity to BV2 microglial cells at concentration of 1–100 µM (Fig. 6a). Second, we evaluated the concentration of GS which could block FXR expression. The results showed that GS blocked FXR at a dose dependent manner (Fig. 6b). In this study, the optimal concentration that could block FXR was 100 µM. Finally, we evaluated whether blockage of FXR could reverse the effect of GAA in LPS-induced BV2 microglial cells. The results showed that blocking FXR could reverse the down-regulation of TNF-α (Fig. 7a) and the up-regulation of BDNF (Fig. 7b) in LPS-induced BV2 microglial cells after GAA treatment.

### FXR Knock-Down Blocked the Effects of GAA in LPS-Stimulated BV2 Microglial Cells

**Fig. 8:**
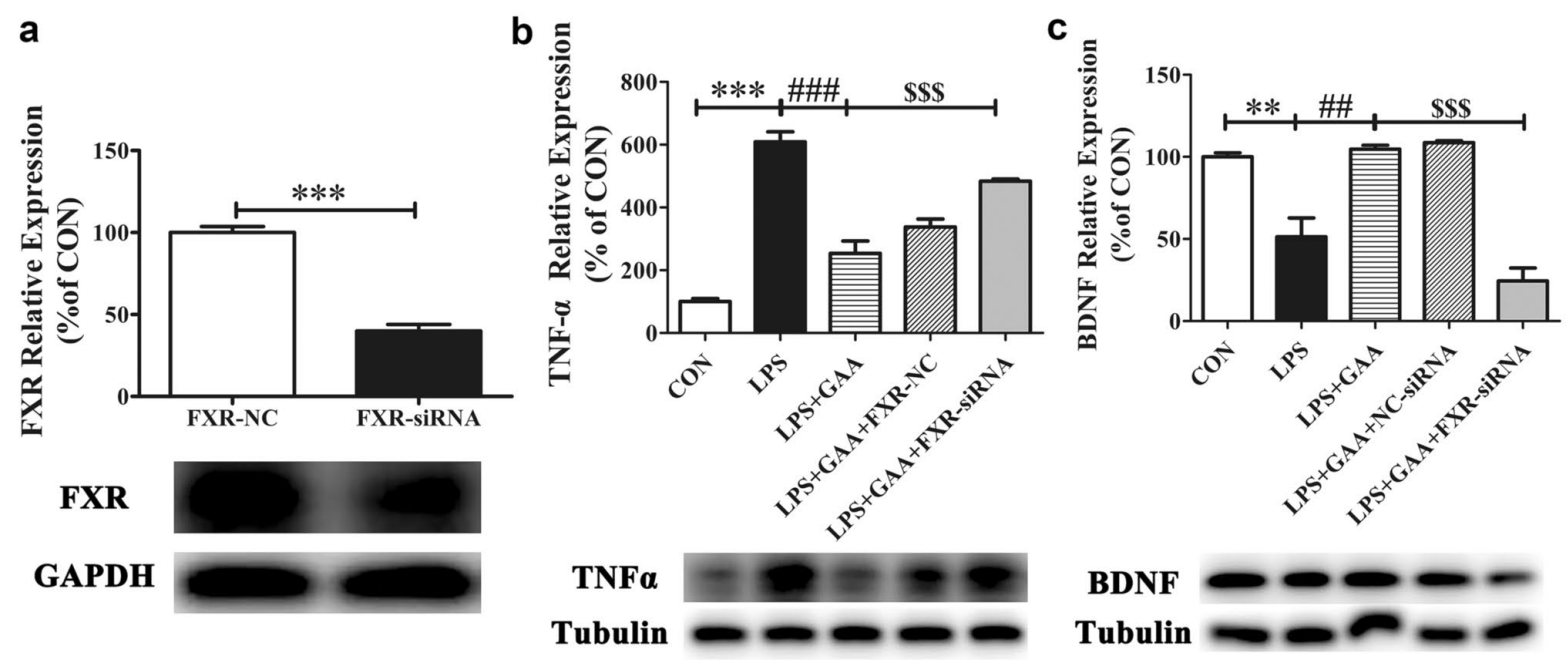
The effects of FXR knock-down on GAA-mediated expression of FXR, TNF-α and BDNF levels in LPS-stimulated BV2 microglial cells. The protein levels of FXR (**a**), TNF-α (**b**) and BDNF (**c**) were detected by Western blot. After normalization to the CON, data from three independent experiments was analyzed using one-way ANOVA followed by post hoc Turkey tests and were presented as Mean ± SEM. (***P < 0.001 FXR-NC vs. FXR-si-RNA; **P < 0.01, ***P < 0.001 LPS vs. CON; ^*##*^*P* < 0.01*, *^*###*^*P* < 0.001 LPS + GAA vs. LPS; ^*$$$*^*P* < 0.001 LPS + GAA + FXR-si-RNA vs. LPS + GAA)

To further determine whether FXR affects the effect of GAA on LPS-stimulated BV2 microglial cells, we chose FXR siRNA to knock down FXR. After 36 h transfection and 24 h cell culture under different treatment, the expression of FXR, TNF-α and BDNF were detected by western blot. The results showed that FXR protein was significantly decreased compared with FXR-NC group (Fig. 8a). Moreover, FXR knock down could significantly reverse the down-regulation of TNF-α (Fig. 8b) and the up-regulation of BDNF (Fig. 8c) in LPS-induced BV2 microglial cells after GAA treatment. These results further demonstrate that GAA could suppress LPS-induced neuroinflammation through activation of FXR in BV2 microglial cells.

## Discussion

We sought to study the effects of GAA on LPS-induced neuroinflammation in BV2 microglial cells and its underlying mechanisms. We found that (1) GAA significantly inhibits LPS-induced BV2 microglial cells proliferation and activation in vitro; (2) GAA promoted the conversion of LPS-induced microglial cells from M1 status to M2 status; 3) GAA prominently attenuated pro-inflammatory cytokines IL-1β, IL-6 and TNF-α and enhanced neurotrophic factor BDNF expression in LPS-induced BV2 microglial cells; (4) GAA reversed LPS-induced FXR down-regulation in BV2 microglial cells; (5) the effects of GAA were blocked after FXR antagonist GS or FXR siRNA treatment in LPS-treated BV2 microglial cells.

Microglia-mediated neuroinflammation is a hall mark of neurodegenerative diseases, including AD, PD, amyotrophic lateral sclerosis (ALS) and MS [6]. Microglia are the resident neuroimmune cells of the central nervous system and play an important role in maintaining homeostasis in normal conditions [22]. In response to injury or stimuli, microglia become readily activated and consequently modulates their phenotypes to adapt the activated state. Accumulating evidence strongly showed that LPS can activate BV2 microglial cells to produce various cytokines, nitric oxide, PGE2, COX2 and iNOS, hence LPS-stimulated BV2 microglial cells were often used as an in vitro neuroinflammation model [23–25]. Previous studies have shown that LPS can induce BV2 microglial cells and brain resident microglia proliferation and activationin vitro and vivo [26–28]. Subsequently, activated microglia releases inflammatory mediators such as TNF-α, IL-1β, IL-6. These inflammatory factors in turn act on microglia and brain and lead to neurodegenerative diseases [29].Cumulative studies have showed that pharmacologic regulation of microglia activation is effective in the treatment of neurodegenerative diseases [30, 31]. Consistent with previous results, our results showed that GAA inhibited LPS-induced BV2 microglial cells proliferationand activation, indicating that it plays an important role in neuroimmune regulation.

Activated microglia, as in macrophages, the phenotypes were heterogeneous, can be divided into either M1 or M2 type [6], which are considered neurotoxic or neuroprotective, respectively [32]. Activation of M1 type microglia releases diverse pro-inflammatory cytokines and oxidative stress-induced free radicals that promotes neuroinflammation and inhibits brain repair. Conversely, activation of M2 type microglia improves brain repair and inhibits neuroinflammation by releasing anti-inflammatory cytokines, neurotrophic cytokines, and enhancing phagocytosis. However, previous studies have shown that most of compounds can suppress neuroinflammation simply by inhibiting M1 microglia activation [10, 33, 34], few compounds can suppress neuroinflammation by promoting the conversion of M1 type to M2 type microglia [35, 36]. Our results showed that GAA treatment significantly inhibited the up-regulation of iNOS and the down-regulation of Arg-1 expression, which indicate that GAA worked as the molecular switch to convert microglia from M1 to M2 type and alleviated inflammation.

Stimulating of BV2 microglial cells by LPS lead to the production of pro-inflammatory cytokines, such as IL-1β, IL-6 and TNF-α, which have been confirmed that could cause neural cell damage, initiate and amplify the inflammatory response, and lead to the development of neurodegenerative diseases [29]; Therefore, the suppression of their production is pivotal for prevention of neurodegenerative diseases [2, 11]. On the contrary, microglia can also secrete anti-inflammatory cytokines and some neurotrophic factors to ameliorate neurodegenerative disease [37, 38], such as BDNF. The expression of IL-4, TGF-β and BDNF were detected in this study, unfortunately we did not discover the any changes of IL-4 and TGF-β in LPS-induced BV2 microglial cells. Previous study had reported that GAA could not decrease the level of TNF-α, IL-6 and IL-1β in the cell culture supernatants of LPS-stimulated primary mouse microglia, but GAA could decrease the expression of TNF-α, IL-6 and IL-1β in the cell lysates of LPS-stimulated primary mouse microglia [39]. Our results showed that GAA significantly attenuated pro-inflammatory cytokines IL-1β, IL-6 and TNF-α and enhanced neurotrophic factor BDNF expression in cell lysates of LPS-stimulated BV2 microglial cells, but not in the cell culture supernatant of LPS-stimulated BV2 microglial cells, which was in consistent with previous findings.

FXR has been extensively studied in liver disease, such as innate hepatic inflammation, cholestatic liver disease and non-alcoholic fatty liver disease (NASH) [17]. Intriguingly, FXR agonist has been tested in clinic trial for treatment of liver disease, demonstrating that FXR has become an attractive target in human metabolic disease. In fact, FXR was not only expressed in liver, gut and kidney [40], but also expressed by immune cells, OPCs and mature oligodendrocytes, like microglia and astrocyte [41]. Previous studies have shown that FXR expression was significantly decreased after LPS stimulation in monocytes [42] and IFNγ stimulation in macrophages [43], which indicated a link between chronic autoimmune inflammation and FXR expression. However, whether or not FXR expression is changed after LPS stimulation in BV2 microglial cells remains unclear. In the present study, our results further confirmed that FXR plays an important role in regulating chronic inflammation.

FXR activation has been proved to confer protection in LPS-induced neuroinflammation in BV2 microglial cells [44, 45]. However, in order to further validate the effect of FXR in LPS-induced neuroinflammation in BV2 microglial cells, GS and FXR-siRNA, were chosen to block FXR in this study. The present study found that GS or FXR-siRNA treatment can significantly reverse the effect of GAA in inhibiting TNF-α and promoting BDNF expression in LPS-induced BV2 microglial cells. These results indicatedthat GAA inhibit LPS-induced neuroinflammation through activation of FXR.

In conclusion, this study demonstrates that GAA suppressed LPS-induced BV2 microglial cells proliferation and activation, promoted the conversion of M1 type microglia to M2 type, inhibited the LPS-induced pro-inflammatory cytokine release, and enhanced the neurotrophic factor BDNF expression. The precise mechanism of GAA in inhibiting LPS-induced neuroinflammation was mainly via activating FXR. Our results have strongly supported that GAA exert an anti-inflammation role in the context of neuroinflammation. Therefore, GAA may be a valuable anti-inflammatory and neuroprotective candidate for the treatment of brain diseases associated with inflammation.

## Declarations

### Data Availability Statement

The data are available from the corresponding author upon reasonable request.
